# National-scale simulation of human movement in a spatially coupled individual-based model of malaria in Burkina Faso

**DOI:** 10.1038/s41598-022-26878-5

**Published:** 2023-01-06

**Authors:** Robert J. Zupko, Tran Dang Nguyen, Amy Wesolowski, Jaline Gerardin, Maciej F. Boni

**Affiliations:** 1 Center for Infectious Disease Dynamics, Pennsylvania State University, University Park, PA 16802 USA; 2 Department of Epidemiology, Johns Hopkins Bloomberg School of Public Health, Baltimore, MD USA; 3 Department of Preventive Medicine and Institute for Global Health, Northwestern University, Chicago, IL USA; 4 Nuffield Department of Medicine, University of Oxford, Oxford, UK

**Keywords:** Computational science, Malaria, Epidemiology

## Abstract

Malaria due to the *Plasmodium falciparum* parasite remains a threat to human health despite eradication efforts and the development of anti-malarial treatments, such as artemisinin combination therapies. Human movement and migration have been linked to the propagation of malaria on national scales, highlighting the need for the incorporation of human movement in modeling efforts. Spatially couped individual-based models have been used to study how anti-malarial resistance evolves and spreads in response to drug policy changes; however, as the spatial scale of the model increases, the challenges associated with modeling of movement also increase. In this paper we discuss the development, calibration, and validation of a movement model in the context of a national-scale, spatial, individual-based model used to study the evolution of drug resistance in the malaria parasite.

## Introduction

The promulgation of the Millennium Development Goals, and specifically Goal 6 to combat HIV/AIDS, malaria, and other diseases, by the United Nations has resulted in a renewed global interest in malaria eradication. However, despite the efforts of the past 20 years, malaria caused by the *Plasmodium falciparum* parasite remains a serious public health concern with 241 million cases and 627 thousand deaths estimated in 2020^1^. One significant barrier to eradication efforts is the evolution of anti-malarial resistance by the parasite, with resistance to the artemisinin components of artemisinin combination therapies (ACTs) being of particular concern^1^. While the primary driver of anti-malarial resistance is the evolutionary pressure applied on the parasite through the use of anti-malarial treatments (e.g., ACTs)^2–4^, a resistant parasite may also appear in a region due to importation though human movement (e.g., temporary travel for work or leisure) or migration (i.e., permanent relocation)^5^.

One means of studying the evolution of anti-malarial resistance—and the impact that various drug policies may have upon it—has been through the use of spatially coupled, stochastic, individual-based models (IBMs) that incorporate components such as: transmission of the parasite, immune acquisition and response, genotype evolution, and drug intervention strategies^6^. By incorporating space and geography in these models, it is possible to evaluate possible drug interventions and observe how geography and population distribution factors in to the development of anti-malarial resistance by the parasite. This insight may allow for new eradication strategies to be developed, as well as proper allocation of resources based upon projected movement patterns. Accordingly, modeling efforts must be accompanied by a model of human movement in order to account for the movement of anti-malarial resistant parasites via infected carriers. However, national scale simulations may incorporate millions of simulated individuals, spread across thousands of cells (representing the simulated space of a region or country), resulting in challenges of model implementation, calibration, and validation.

Since human movement and migration has been linked to malaria transmission and the migration of drug resistant genotypes^5,7^, incorporation of human movement is a critical component of such IBMs. The two common mathematical models of human movement used in studies of the Sub-Saharan Africa (SSA) region are gravity and radiation models^8,9^. Gravity models are constructed with the assumption that movement rates between two points (e.g., cities) increase in relation to the size of the point populations and decrease with the square of the distance between term; similar to physical laws of gravitation. Gravity models may also be modified with functions that account for other parameters, such as mode and cost of travel. Likewise, radiation models draw their inspiration from physical processes (i.e., particle diffusion), but differ from gravity models in that the underlying assumption is that individuals move outward from their origin and are “absorbed” by a given destination, within a given distance, with a probability that is proportionate to the population of a given destination.

**Figure 1:**
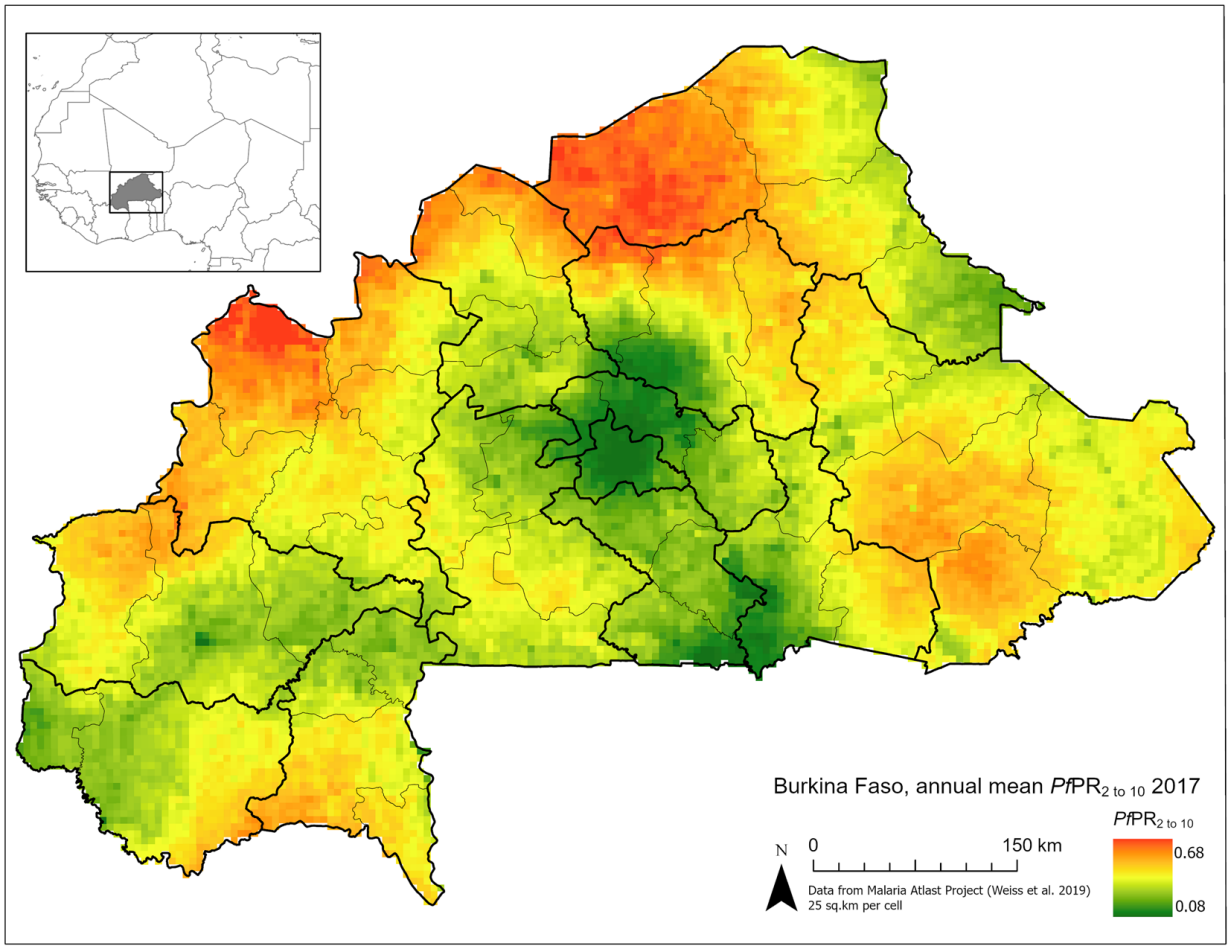
Prevalence of *P. falciparum* malaria in Burkina Faso based the Malaria Atlas Project projections for 2017^10^. Map prepared by the authors using ArcGIS Pro (version 2.9.3, https://www.esri.com/en-us/arcgis) using administrative boundaries from the World Bank Group^11,12^, and prevalence data from the Malaria Atlas Project^10^.

In this study we examine the data and mathematical models of human movement in Burkina Faso. Burkina Faso is a landlocked country in western SSA with endemic malaria (i.e., persistent transmission of the infection in the population) (Fig. 1), which is the leading cause of hospitalization in the general population (45.8% of cases) as well as for children under five (48.2% of cases)^13,14^. During periods of seasonal rainfall, the *P. falciparum* prevalence rate in the two- to ten-year-old population (called *Pf*PR_2-10_) may increase significantly in relation to the annual mean *Pf*PR_2-10_ which ranges from 8.0 to 67.6% based upon 2017 estimates^10^. The primary first-line therapy for uncomplicated malaria are ACTs such as artemether-lumefantrine (AL), which has been recommended since 2005 in accordance with WHO guidelines^14^. ACTs are used in conjunction with other malaria intervention programs such as: insecticide-treated mosquito nets (ITNs), indoor residual spraying (IRS), intermittent preventative treatment of pregnant women (IPTp), and seasonal malaria chemoprophylaxis^15^. As a result of ACT usage and these interventions, the general trend since 2005 has been a reduction in the prevalence of malaria^16^.

**Figure 2:**
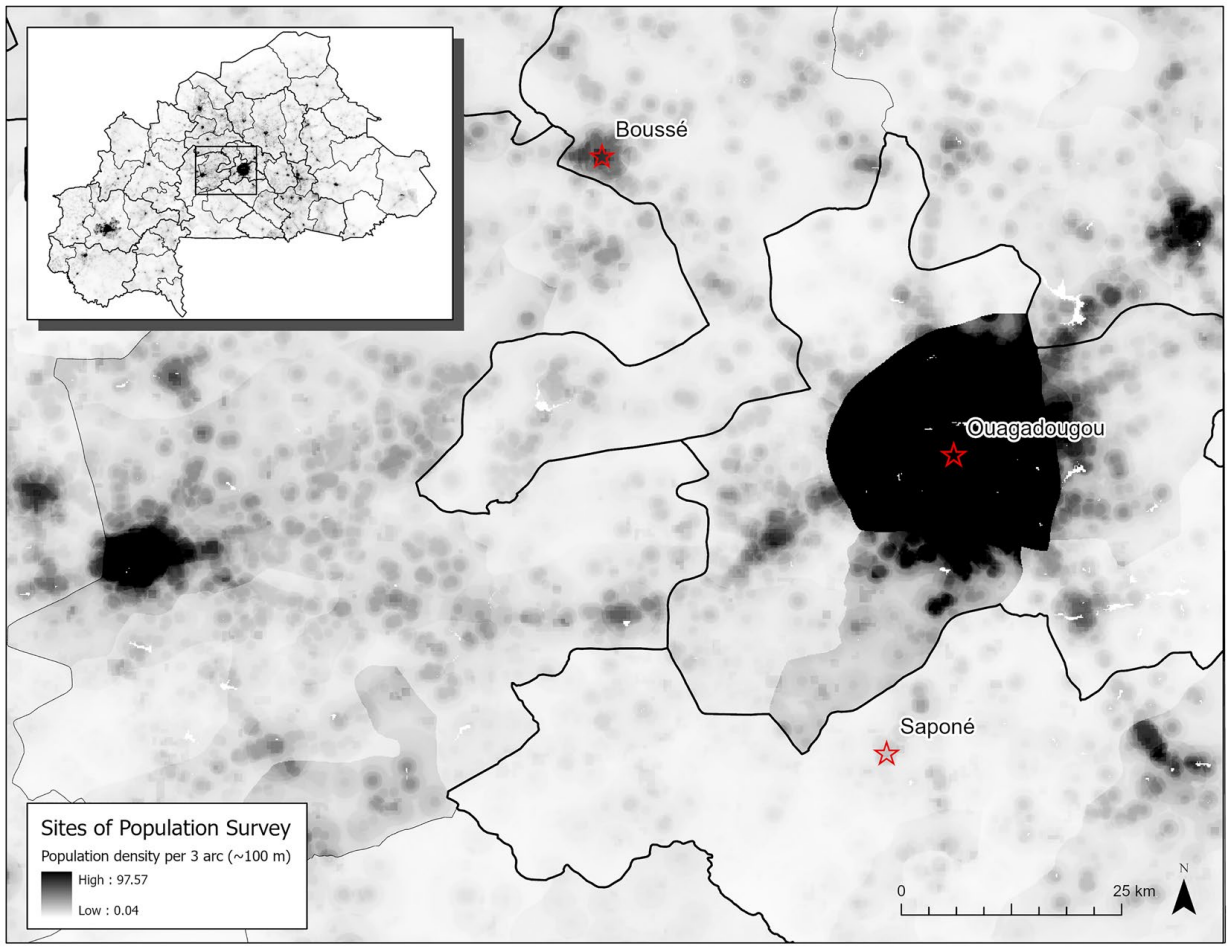
Sites of movement survey (red stars) by Marshall et al.^17^ overlaid population density from WorldPop^18^ at a with a resolution of 3 arc. White areas indicate no population density due to water features, while progressively darker shades indicate higher population density. Note the high population density of the region surrounding the survey site in the capital, Ouagadougou in comparison to the agricultural and trade hub Boussé, or agricultural village of Saponé. Map prepared by the authors using ArcGIS Pro (version 2.9.3, https://www.esri.com/en-us/arcgis) using administrative boundaries from the World Bank Group^11^ and population data from WorldPop^18^.

Surveys of human movement and migration in Burkina Faso are limited^17,19–22^, with Marshall et al.^17^ being the most recent. In their study, three sites were selected: the capital Ouagadougou in Kadiogo province, the agricultural and trade hub Boussé in Kourweogo province, and the agricultural village Saponé in Bazega province (Fig. 2). All sites were sampled during the rainy season of July 2011, with the number of trips recorded based upon the source and destination. Despite being the most recent quantitative study performed, some limitations are present. First, the section of survey sites is clustered around Ouagadougou, the centrally located capital of Burkina Faso. Second, complete coverage of travel to all provinces in Burkina Faso was not captured. Finally, the timing of the survey during the rainy season likely also introduces biases due to seasonal migration patterns.

However, a sufficient quantity of data was collected by Marshall et al.^17^ that it was possible for the authors fit the data using a gravity model and radiation model of movements^8^. Upon completion of model fit, the authors concluded that a gravity model with a power-law distance kernel had the most predictive power in relation to the observed movement patterns. This is consistent with previous studies in SSA that found that radiation models under predict travel compared to gravity models^9^. However, in the context of SSA it has been suggested that countries may follow unique patterns that are not perfectly explained by either the gravity or radiation model in their unmodified forms^9^.

## Results

### Mathematical model

The general success and application of gravity models for human movement and migration in SSA^8,9^, coupled with the recent work by Marshall et al.^8,17^, motivated us to start with the modified gravity model suggested:

1 $$ Pr\left( {j{|}i} \right) \propto Pop_{j}^{{\uptau }} k\left( {d_{i,j} } \right) $$

2 $$ k\left( {d_{i,j} } \right) = \left( {1 + \frac{{d_{i,j} }}{{\uprho }}} \right)^{{ - {\upalpha }}} $$

where ${\text{Pr}}(j|i)$ describes the probability of travel from the source *i* to the destination *j*, given the product of the population of *j* raised to *τ* (Eq. 1), and the distance kernel (Eq. 2), which takes the form of a power law containing the scale parameter *ρ*, and the power-law parameter *α*.

Two limitations are present in the model constructed by Marshall et al.^8^ First, the model presumes that an entire destination (i.e., destination city) can be treated as a point, resulting in an incongruence when used in the context of a grid-based landscape. Second, is the lack of consideration for the time, distance, or complexity in traveling to a given destination. This is particularly important in the context of malaria interventions since rural communities may lack local medical resources, necessitating travel to seek care^5,23^. Accordingly, this limitation can be addressed by capturing the difficulty associated with travel through the use of a friction surface, which quantifies the ease or difficulty in traversing surfaces (e.g., road types) or natural barriers such as mountainous terrain^24^. An alternative is the use of a travel time map (or surface) which estimates the time to reach the nearest city (or high-density urban area) from a given location on the map^25^. Since travel time maps typically annotate the travel time within an urban environment as zero, this also addresses the incongruence that arises since urban boundaries are delineated travel times of zero.

Accordingly, the probability of movement can be adjusted to use travel times as follows:

3 $$ Pr({\text{j}}|{\text{i}})\prime = \frac{Pr(j|i)}{{\left( {1 + t_{i} + t_{j} } \right)}} $$

where ${\text{Pr}}(j|i)\prime$ describes the new probability of travel from source *i* to the destination *j* following the division of the original probability ${\text{Pr}}(j|i)$ by the sum of one plus travel time to the nearest city of the source *t*_*i*_ and destination *t*_*j*_. Incorporation of both the source *t*_*i*_ and destination *t*_*j*_ in the denominator of Eq. (3) ensures accounting for the costs of indirect travel routes. This has the effect of biasing the model towards destination cells that are located in (i.e., travel time of zero) or near (i.e., low travel time to the nearest city) cities, but still allowing travel between rural locations, or from the city to a rural location. Note that since within city travel is unpenalized (i.e., travel time of the source and destination is zero), during model calibration, care must be taken to ensure that the number of trips (e.g., within a city or province, or between cities or provinces) are properly accounted for. If necessary, ${\text{Pr}}(j|i)\prime$ can be adjusted by dividing by a penalty parameter, *p*, in the event the model is biased towards movement remaining within a given area.

### Evaluation of implemented model

**Figure 3:**
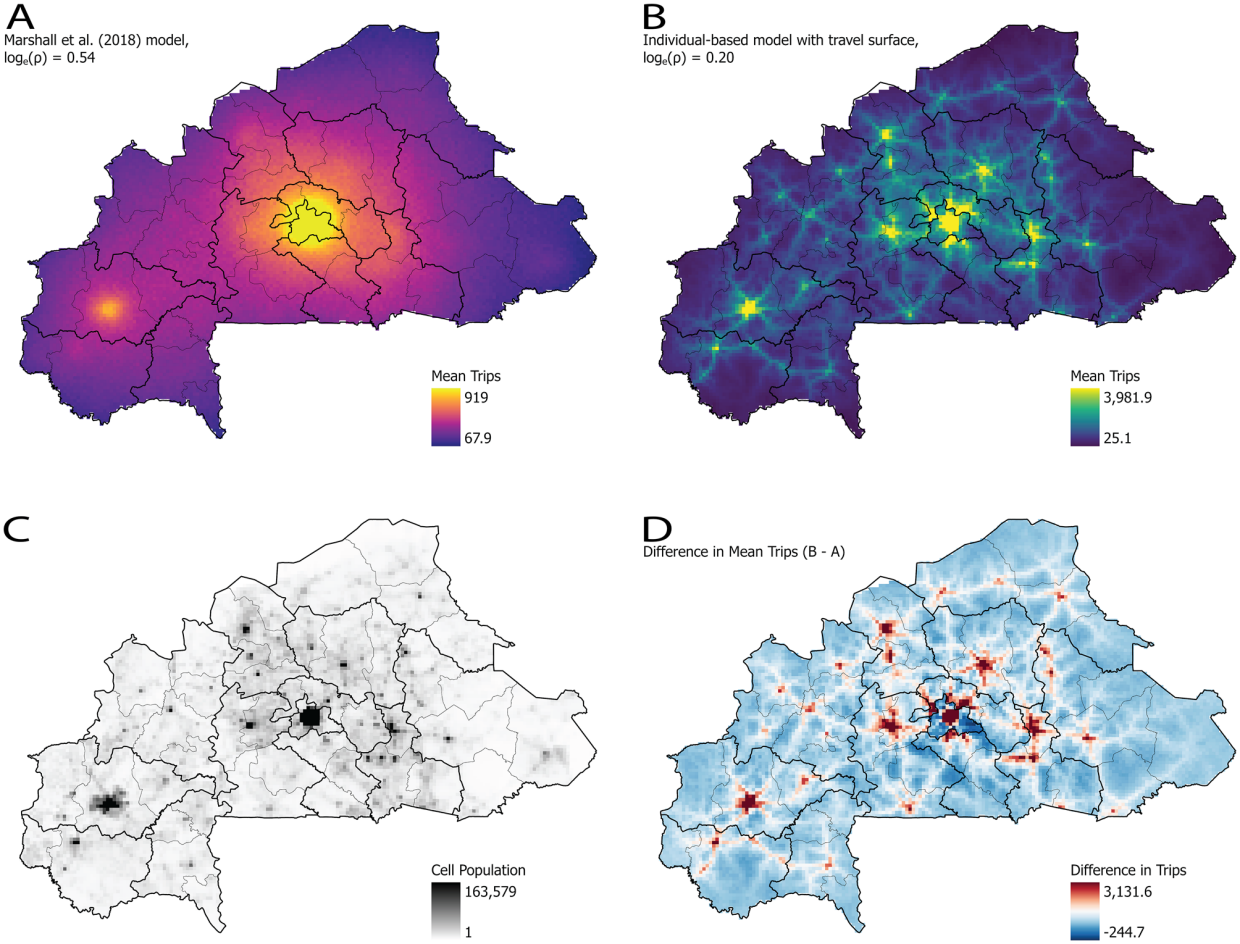
Comparison of the gravity model with kernel function parameterized by Marshall et al.^8^ versus the mathematical model with travel surface and parameterization described, as implemented in the individual-based model. (**a**) The mean number of trips for ten replicates to the destination cell in a single month using the parametrization prepared by Marshall et al.^8^ (**b**) The mean number of trips for ten replicates to the destination cell in a single month using the mathematical model with travel surface and parametrization described in this manuscript. (**c**) The population distribution of Burkina Faso from WorldPop^18^ aggregated into the same 5 km-by-5 km cells used for movement. (**d**) The raster algebra difference between the mean number of trips to the destination cell from (**a**,**b**); note that the Marshall et al.^8^ model tends to project higher movement to rural areas (negative values in blue tones) compared to (**b**) which is shifted towards the areas of higher population using the method described here (positive values in red tones). Furthermore, the movement is less diffused compared to (**a**). However, the lack of diffusion results in movement being better aligned with the concentrations of population and transportation networks compared to (**a**) despite the total number of trips being similar. Maps prepared by the authors using ArcGIS Pro (version 3.0.3, https://www.esri.com/en-us/arcgis) using administrative boundaries from the World Bank Group^11^ and data from the simulation described.

The mathematical model developed—that is Eqs. (1–3)—was implemented as part of a stochastic IBM developed for Burkina Faso^6,26^, and parameterization of the movement equations was performed by first estimating the best fit for the ρ based upon the $log_{e} \left( \rho \right)$ from the range of values suggested by Marshall et al.^8^, with $log_{e} \left( \rho \right) = 0.20$ having the best fit to the survey data^17^. Next, the inferred values $\alpha = 1.27$ and $\tau = 1.342$, as fit by Marshall et al.^8^, were used in an IBM of Burkina Faso for one month with a population of approximately 19 million individuals, and all travel between cells was logged. The cellular movement of the simulation was aggregated to the province level and compared to the Marshall et al.^17^ survey data. Initial calibration results revealed a model bias in Kadiogo province—containing the capital Ouagadougou—due to individual movement remaining within the province as a result of the high population relative to the rest of the country. This was corrected by dividing Pr’ by the model penalty $p = 12$ when the cell is within Kadiogo province, where the penalty value was found though model fitting. As a result, it was found that the movement probability ${\text{Pr}}(j|i)\prime$, coupled with a model penalty applied to Kadiogo Province, produces a reasonable replication of the source to destination movement in regions of Burkina Faso with high, geographically dispersed populations (Fig. 3).

Evaluation of the projected movement was performed using the mean of ten replicates for six different configurations (i.e., Marshall et al.^8^ model with published calibration, biased towards shorter travel, and our model fit; along with the model described here and the three parameterizations), all of which produced a similar number of trips (1,898,288 ± 555) (see Supplemental Information 1, Fig. S1). When comparing the gravity model and parameterization suggested by Marshall et al.^8^ it is clear that the projected population movement (Fig. 3a) is diffuse with smaller population centers being bypassed by individuals in favor of Ouagadougou. However, the results of the mathematical model with travel surface (Fig. 3b) show trips that are aligned with the population distribution of Burkina Faso (Fig. 3c). Furthermore, the gravity model appears to under project the number of trips to major population centers while over projecting the number of trips to areas of low population. (Fig. 3d).To further contrast the differences in travel, an additional forty replicates (total *n* = 50) were run for the Marshall et al.^8^ model with published calibration, and the model described here with the best model fit (see Supplemental Information 2). Both models produce similar results for low population cells (i.e., < 1000); however, as expected, the models diverge significantly as the population increases (see Supplemental Information 1, Fig. S4).

**Figure 4:**
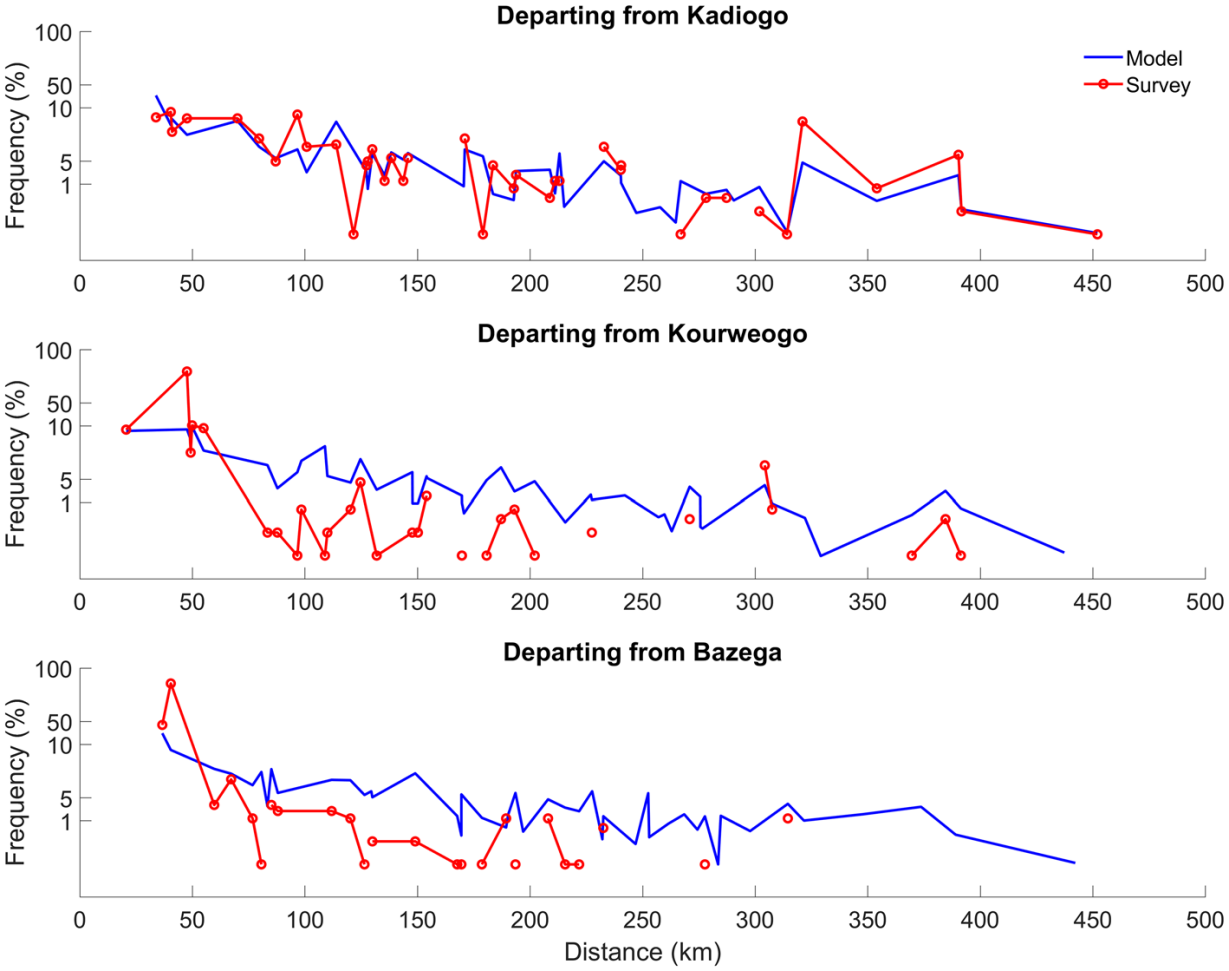
Trips from Kadiogo providence compare favorably with the Marshall et al.^17^ survey on the basis of distance traveled. However, the limited survey data for Kourweogo and Bazega pose a challenge to model validation, although the simulated results are generally consistent with the survey data.

Finally, when the total number of trips is compared to the distance traveled, the results are also comparable to the survey data (Fig. 4). However, given the sparsity of survey data for Kourweogo and Bazega (Fig. 4), the projected short distance travel is consistent with the survey data and long-distance travel—which has a lower overall frequency resulting in fewer survey respondents—is generally consistent with similar travel out of Kadiogo province. To reduce the effects of the long model tails seen in the bottom two panels of Fig. 4, an alternative value of $log_{e} \left( \rho \right) = 0.45$ was selected. The difference was small with slightly less travel to population centers compared to the parameterization suggested by Marshall et al. or the $log_{e} \left( \rho \right) = 0.20$ value (see Supplemental Information 1, Figs. S1, S2).

## Discussion

With individually-based models (IBMs) designed for, and calibrated to, epidemiological models of malaria, an appropriate model of movement by individuals is necessary due to the role that human movement and migration plays in the spread of anti-malarial resistance. However, the development, calibration, and validation of movement models, particularly at the national scale, remains a challenge. These challenges can be further compounded by the lack of quantitative data available for a given country of interest. Despite this, IBMs and mathematical modeling more broadly, still play an important role in malaria control with national scale models offering possible insights for surveillance and drug policy response^27^.

Despite these challenges, geospatially coupled malaria models are adventitious in that they can produce projections for where anti-malarial resistance may arise, allowing surveillance efforts to focused on narrow geographic scopes. An important application of malaria IBMs is evaluation of how changes in drug policy (e.g., changing the partner drug in an ACT or introducing multiple first-line therapies^6,26,28,29^) impact the emergence of drug resistance by the parasite, and the connection between antimalarial drugs and de novo emergence of resistance has been well established^2–4^, accordingly it is not a matter of if drug resistance will emerge, but rather when. However, the role that human migration plays in introducing an anti-malarial resistant parasite to a region remains unclear.

A standard movement model, or a regionally calibrated movement model such as the one developed by Marshall et al.^8^, is not guaranteed to meet the needs of a model developer “out of the box.” While a published parameterization may offer a useful starting point for model developers, additional algorithm development, parameterization, and calibration steps are necessary to ensure that the model is appropriate for expected movement in a region, and the goals of the model. As this paper has demonstrated, one approach to improving model fidelity is through the use of a travel time map. Although, as always, model developers should be diligent during the calibration and validation process to ensure that model outputs make sense in the context of quantitative and qualitative data that is available.

While the introduction of an anti-malarial resistant *P. falciparum* parasites to a region through human movement and migration is just one mechanism by which resistance can appear; it is necessary that IBMs modeling malaria with epidemiological goals properly account for it. As this paper has demonstrated, it is possible to “scale-up” mathematical models to be utilized in national-scale IBMs; however, performance, calibration, and validation are all challenges that require careful investigation. Furthermore, the availability of data for a region of study can place limitations on the extent of validation that is possible, necessitating some caution in the claims that can be made by simulating specific scenarios.

## Methods

### Implementation

The mathematical model described was implemented using C++, in an IBM previously developed by Nguyen et al.^6^ During execution of the simulation, individual movement is based upon a decision to (i) leave the current location, and (ii) where to travel to at the end of each timestep—representing one day in this instance—as part of the following operations. First, geographic data (i.e., population, travel times, etc.) is read into the simulation state. On each simulation timestep the population events (e.g., infections, births, deaths, etc.) are processed for each cell in the simulation, and the number of individuals in the cell is determined. Next, the individuals in the cell are selected for travel based upon the overall probability of movement in a given year (see Calibration and Validation). This is followed by the calculation of ${\text{Pr}}(j|i)\prime$ for the current cell *i* to each other cell in the simulation, resulting in a vector from which a random, multinomial draw is performed for each individual selected for movement, indicating their destination cell. Individuals are then moved to their destination cell at the end of the time step—ensuring that the population of the cells remain synchronized—and a timer is set to indicate if, and when, they will return to their original cell. If no timer is set, then they remain in their current cell.

The incorporation of the movement model resulted in a number of time and space complexity challenges. Analysis of the source code suggests an algorithmic complexity of $O\left( {n^{3} } \right)$ with a space complexity of $O\left( n \right)$. In order to ensure efficient use of resources, static memory along with the singleton design pattern was used to eliminate the need to reload or copy geographic data, which is represented in memory as either a matrix or a flat array. While it was originally hypothesized during development that the probability of movement could be calculated once at model initialization based upon the initialization parameters, doing so resulted in artifacts appearing in simulation movement over time. However, when the probability is calculated during each timestep (i.e., in a manner consistent with population growth in the simulation) the model performed as expected suggesting that caching of calculated movement probabilities is unlikely to be possible. This suggests that most optimizations are likely to come from careful programming and code organization to minimize the number of times that movement probabilities need to be calculated per timestep.

Finally, in preparing the spatial data used for the calibration of Burkina Faso, the limiting factor of the spatial resolution is the cellular size of the reference *Pf*PR_2-10_ values provided by the Malaria Atlas Project^10^, resulting in a cell size of 25 km^2^. This results the approximately 273,000 km^2^ of Burkina Faso being converted into 10,936 pixels (or cells). Initial population data comes from WorldPop^18^, which was aggregated using ArcMap 10.7.1–25 km^2^ cells, resulting in pixel level populations ranging from 1 to 206,607 individuals per cell. The travel time are derived from Malaria Atlas Project travel time to cities^25^, with the original 1 km^2^ cells aggregated on the basis of the cellular mean to 25 km^2^.

### Calibration and validation

Model calibration begin by fitting the scale parameter *ρ*, based upon the range of values suggested by Marshall et al.^8^ who noted that improvements upon it may be possible. To fit *ρ*, a “synthetic survey” was programmed using MATLAB 2019b. The script approximated the sampling of the Marshall et al.^17^ by drawing a destination for each trial from a given province (i.e., Kadiogo, Kourweogo, and Bazega) by applying the gravity model fit by Marshal et al.^8^ (Eqs. 1 and 2). The distance ($d_{i,j}$) was based upon the distance between the centroid of each combination of providence, calculated using ArcMap 10.7.1, while the population of the province ($Pop_{j}$) was calculated using WorldPop data^18^. The TLAB script then iterated though the iterating through values 0.05 to 1.8 by steps of 0.05 for $log_{e} \left( \rho \right)$ taking a random draw from source *i* to destination *j* based upon a random draw of samples equal to the number of survey participants traveling from each province (Kadiogo *n* = 403, Kourweogo *n* = 503, Bazega *n* = 387). This was repeated for a total of 1,000 trials for each value to ensure sufficient statistical power.

The results of the synthetic survey were then compared to the survey data from Marshall et al.^17^ and the number of matches and inter-quartile ranges (IQR) were compared. While Marshall et al.^8^ suggests $log_{e} \left( \rho \right) = 0.54$ as the best fit, the value $log_{e} \left( \rho \right) = 0.20$ was selected since it offered the most IQR matches along with a low mean squared error. As an alternative, $log_{e} \left( \rho \right) = 0.45$ was noted to evaluate the impact of biasing the model fit towards the longer trips, with minimal impact upon the overall results (see Supplemental Figs. S1 and S2). To further validate the use of $log_{e} \left( \rho \right) = 0.20$, the parameter was used as a simulation parameter in the IBM, with the frequency of trips and distanced traveled tabulated. The simulation results compared favorably to the survey data, although some gaps in the survey data preclude a complete one-to-one comparison of simulation results to survey data.

Next, the model bias resulting from travel departing from Kadiogo province—containing the capital of Burkina Faso—remaining within the province, was corrected by determining the penalty *p* to be applied to ${\text{Pr}}(j|i)\prime$. Based upon a parameter space search in which simulation was run with varying values for *p* a 12-fold intra-province travel penalty was found to be sufficient to eliminate the bias. This was again validated by comparing simulation results against the survey data.

Finally, the precise number of trips was calibrated starting with the finding by Marshall et al.^17^ that about 29.1% of the population of Burkina Faso travels on a yearly basis with a mean of 3.42 trips. This suggests a daily circulation rate (i.e., the daily probability of an individual traveling) of 0.002727 for travelers leaving their home province. However, since the model (and simulation) allows for intra-province travel, it is necessary to scale this value up to ensure that inter-provincial travel is correct. Using the simulation with the aforementioned calibration parameters, individual movement was tracked in the model and various circulation rates trialed until the national level annual rates (29.1%) were matched by a daily circulation rate of 0.00336. As a result of this rate, approximately 19% of all trips remain within a province (i.e., travel between cells in the same province) while the remainder leave. A final set of trials were performed using the simulation and the calibrated parameters, resulting in simulated national-scale movement that is largely consistent with survey data (Fig. 3).

## Supplementary Information

Supplementary Information 1.

Supplementary Information 2.

## Data Availability

The datasets generated and/or analyzed during the current study are available in the malariaibm-movement-BurkinaFaso-2022 repository, https://github.com/bonilab/malariaibm-movement-BurkinaFaso-2022. The source code for the simulation and/or data analysis can be found under the Source directory. The raster data produced by the simulation and used to produce Fig. 3a,b can be found under the Data/GIS directory while processed survey data from Marshall et al.^17^ and intermediary simulation results used to produce Fig. 4 is found under Data/Movement.
