# Supplementary material for: National-scale simulation of human movement in a spatially coupled individual-based model of malaria in Burkina Faso
Source: Sci Rep. 2023 Jan 6;13:321. doi: 10.1038/s41598-022-26878-5 (PMC9822930; doi:10.1038/s41598-022-26878-5)
Supplement: Supplementary file 1 — Supplementary Information 1. [file 41598_2022_26878_MOESM1_ESM.docx]

Figure S1. Comparison of the projected number of trips by movement model and $\boldsymbol{l}\boldsymbol{og}_{\boldsymbol{e}}\left( \boldsymbol{\rho} \right)$ parametrization. (Top) The results using the mathematical model described in this manuscript and the $\boldsymbol{l}\boldsymbol{og}_{\boldsymbol{e}}\left( \boldsymbol{\rho} \right)$ values of (a) 0.54 as suggested by Marshall et al. (2018), (b) 0.45 which biases the model towards shorter travel distances, and (c) the revised value of 0.2. Note that the results are largely similar between the three parameterizations, although dramatically different compared to (Bottom) the results of the gravity model with kernel function with the same $\boldsymbol{l}\boldsymbol{og}_{\boldsymbol{e}}\left( \boldsymbol{\rho} \right)$ values of (d) 0.54, (e) 0.45, and (f) 0.2. Note as the $\boldsymbol{l}\boldsymbol{og}_{\boldsymbol{e}}\left( \boldsymbol{\rho} \right)$ value gets smaller with the gravity model, the travel also becomes less diffuse (bottom row, left to right). Maps prepared by the authors using ArcGIS Pro (version 3.0.3, https://www.esri.com/en-us/arcgis) using administrative boundaries from the World Bank Group (2018) and data from the simulation described.


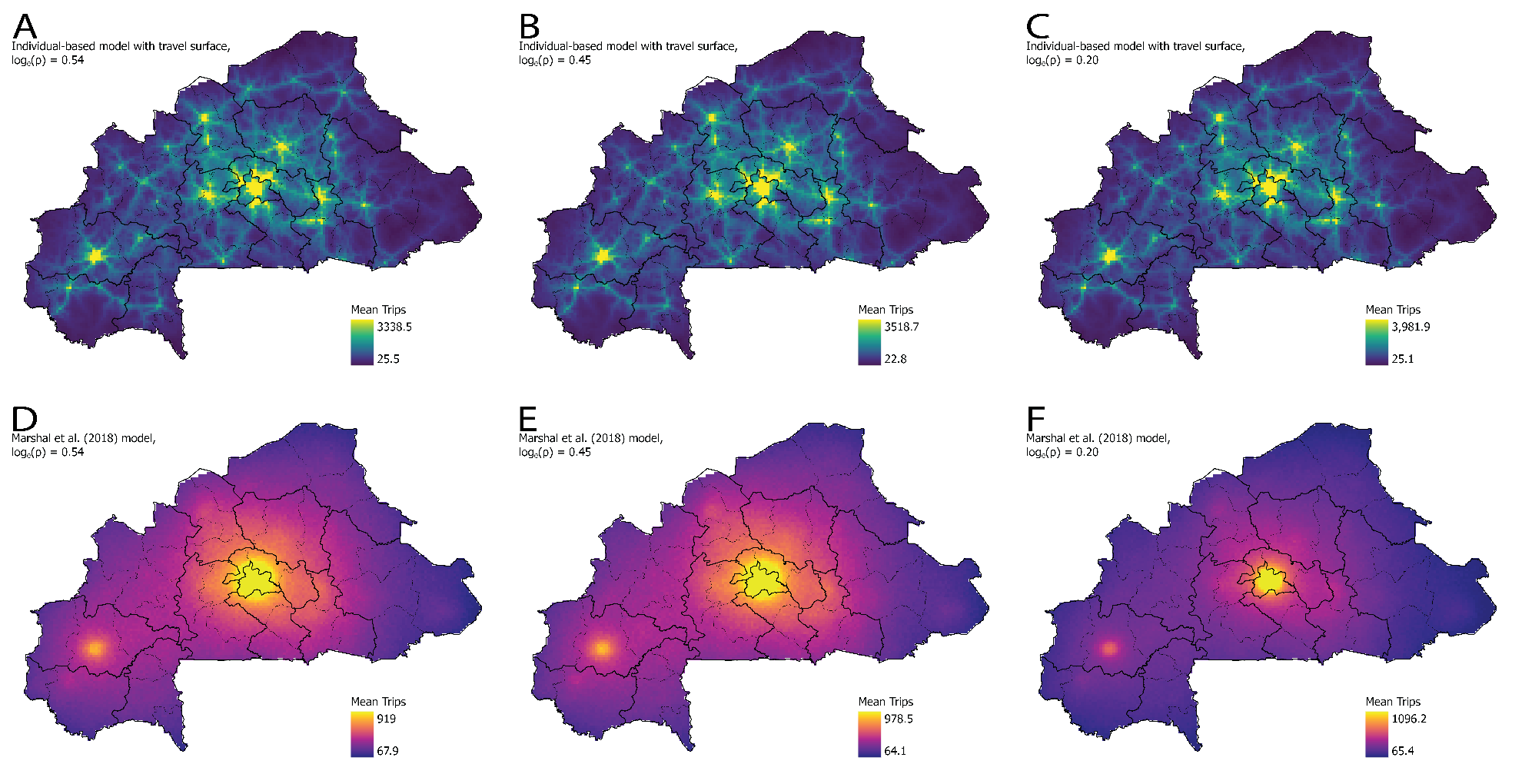


Figure S2. Raster algebra difference between the values models. (a) The difference between our mathematical model using a $\boldsymbol{l}\boldsymbol{og}_{\boldsymbol{e}}\left( \boldsymbol{\rho} \right)\boldsymbol{=0.2}$ and the gravity model with kernel function fit by Marshall et al. (2018). (b) The difference between our mathematical model and the gravity model when both use the $\boldsymbol{l}\boldsymbol{og}_{\boldsymbol{e}}\left( \boldsymbol{\rho} \right)\boldsymbol{=0.2}$ parametrization. Note that the differences between (a) and (b) are fairly minimal, although (b) suggests fewer trips taking place around Ouagadougou. (c) The difference between the mathematical model when $\boldsymbol{l}\boldsymbol{og}_{\boldsymbol{e}}\left( \boldsymbol{\rho} \right)\boldsymbol{=0.2}$ versus $\boldsymbol{l}\boldsymbol{og}_{\boldsymbol{e}}\left( \boldsymbol{\rho} \right)\boldsymbol{=0.54}$. (d) The difference between the mathematical model when $\boldsymbol{l}\boldsymbol{og}_{\boldsymbol{e}}\left( \boldsymbol{\rho} \right)\boldsymbol{=0.2}$ versus $\boldsymbol{l}\boldsymbol{og}_{\boldsymbol{e}}\left( \boldsymbol{\rho} \right)\boldsymbol{=0.45}$. As with (a) and (b) the difference that results from the change in $\boldsymbol{l}\boldsymbol{og}_{\boldsymbol{e}}\left( \boldsymbol{\rho} \right)$ is fairly minimal. Maps prepared by the authors using ArcGIS Pro (version 3.0.3, https://www.esri.com/en-us/arcgis) using administrative boundaries from the World Bank Group (2018) and data from the simulation described.


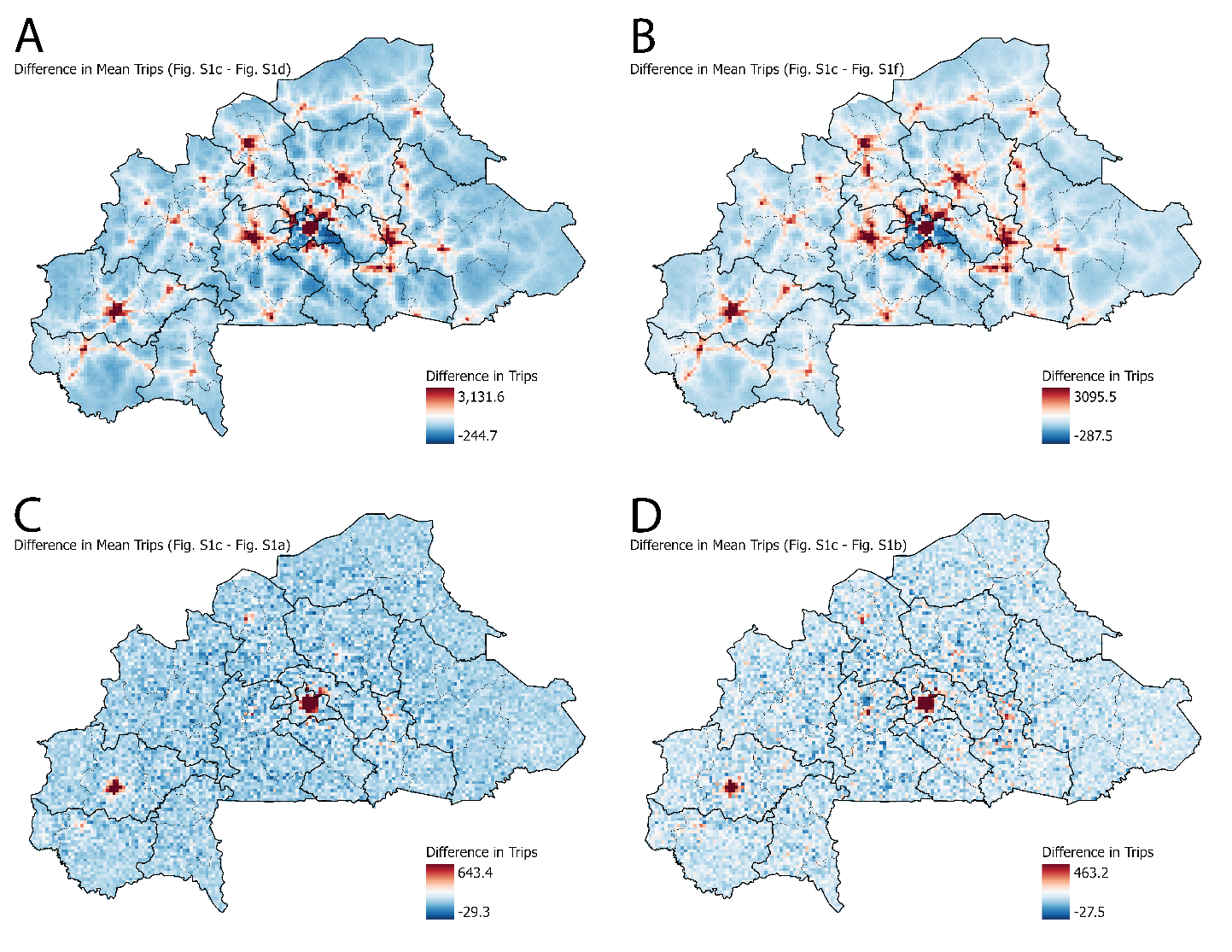


Figure S3. Comparison of the mean projected 580Y frequency count (*n =* 50) between the model described in this manuscript (top row) versus gravity model with kernel function (bottom row). Note that while there is a high degree of stochasticity present in the models, the general trend is for the gravity model with kernel function to result in a more diffuse 580Y frequency compared to the incorporation of a travel surface. (a) The model with travel surface and same parameterization as Marshall et al. (2018). (b) The model with travel sources and biased towards shorter travel distances. (c) The model with travel surface and tuned parameterization, used as the basis for Zupko et al. (2022). (d) Projected 580Y frequency using the movement model by Marshall et al. (2018). (e) The results when adjusted for shorter travel times. (f) The results when using the same tuned $\boldsymbol{l}\boldsymbol{og}_{\boldsymbol{e}}\left( \boldsymbol{\rho} \right)$ value as (c). Maps prepared by the authors using ArcGIS Pro (version 2.9.3, https://www.esri.com/en-us/arcgis) using administrative boundaries from the World Bank Group (2018) and data from the simulation described.


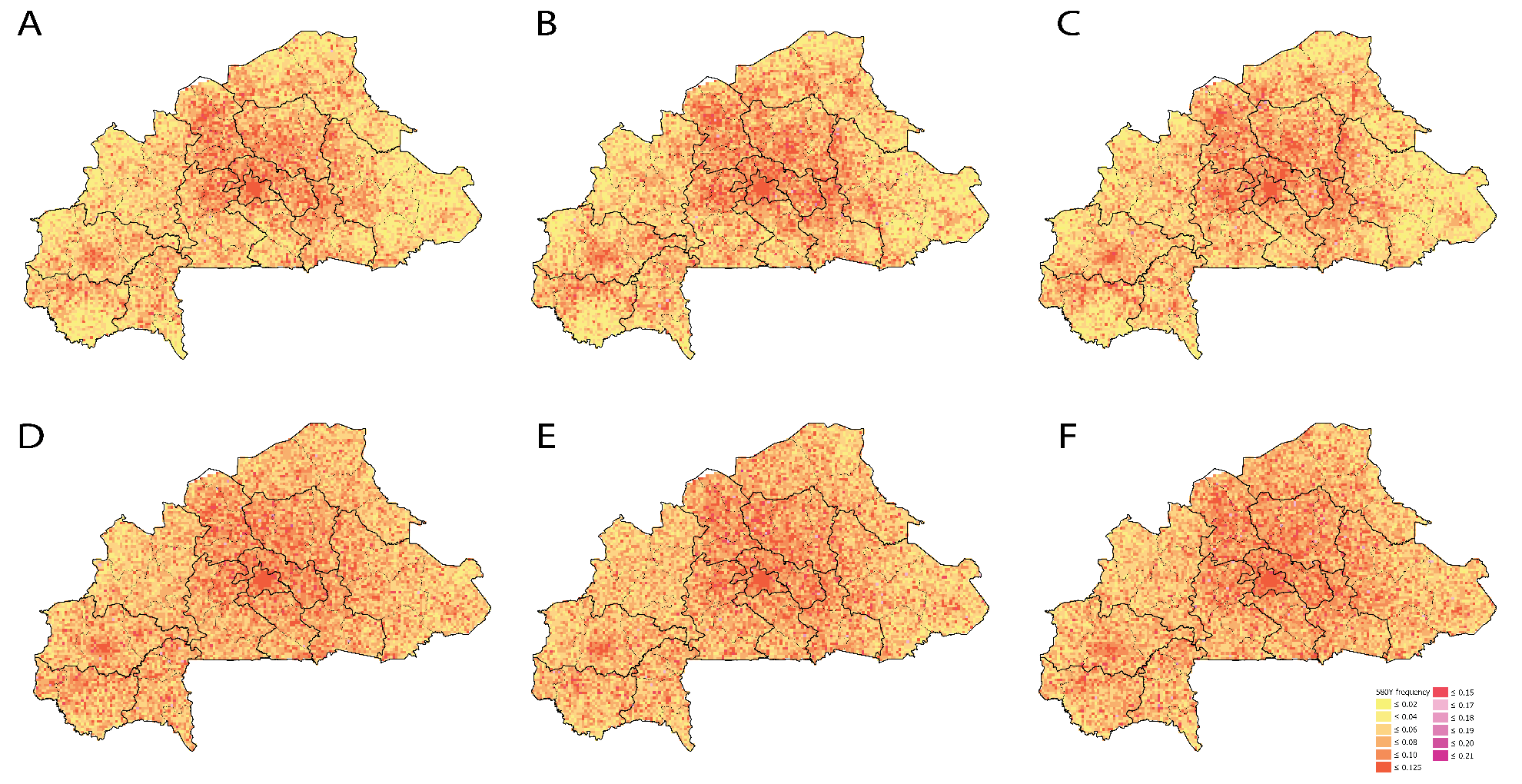


Figure S4. Comparison of the number of trips from the model by Marshall et al. (2018) (dark circles) to the best fit for the mathematical model with travel surface (gray circles) over fifty replicates with the lines representing the standard deviation. The cells for analysis and plotting where determined by first selecting cells with a population greater than 7,000; followed 1 in every 100 cells thereafter. Note that while the model developed by Marshall et al. (2018) produces a gradual increase in the number of trips, the mathematical model with travel surface biases individuals to the closest major population center. Clustering for high population centers is present and an apparent outliner attributable due being on the outskirts of Ouagadougou.


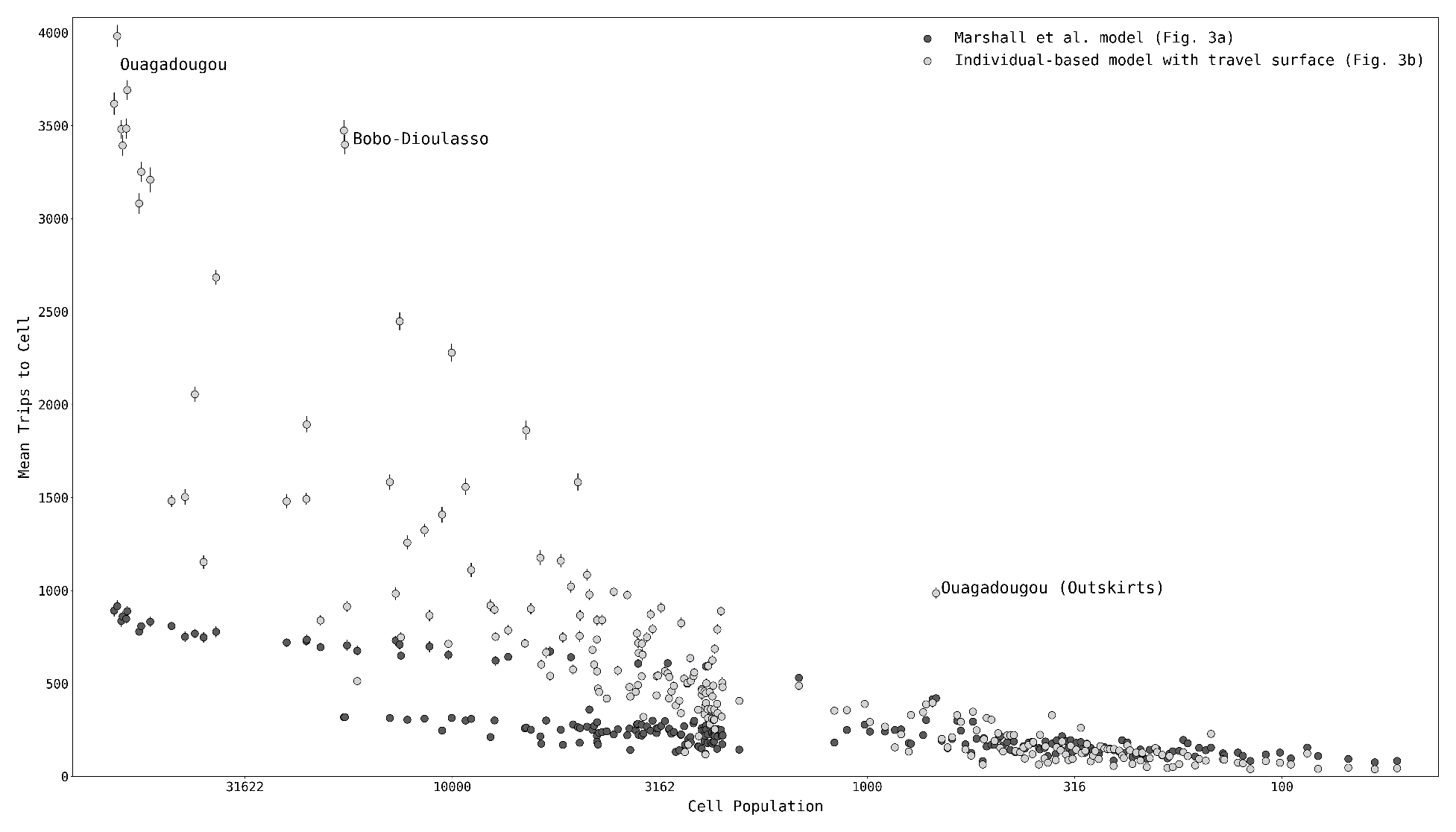


Figure S5. Binned population distribution of Burkina Faso used in preparation of Figure S4. Note that a significant amount of the cells is generally of low population. Map prepared by the authors using ArcGIS Pro (version 3.0.3, https://www.esri.com/en-us/arcgis) using administrative boundaries from the World Bank Group (2018) and population data from WorldPop (2018).


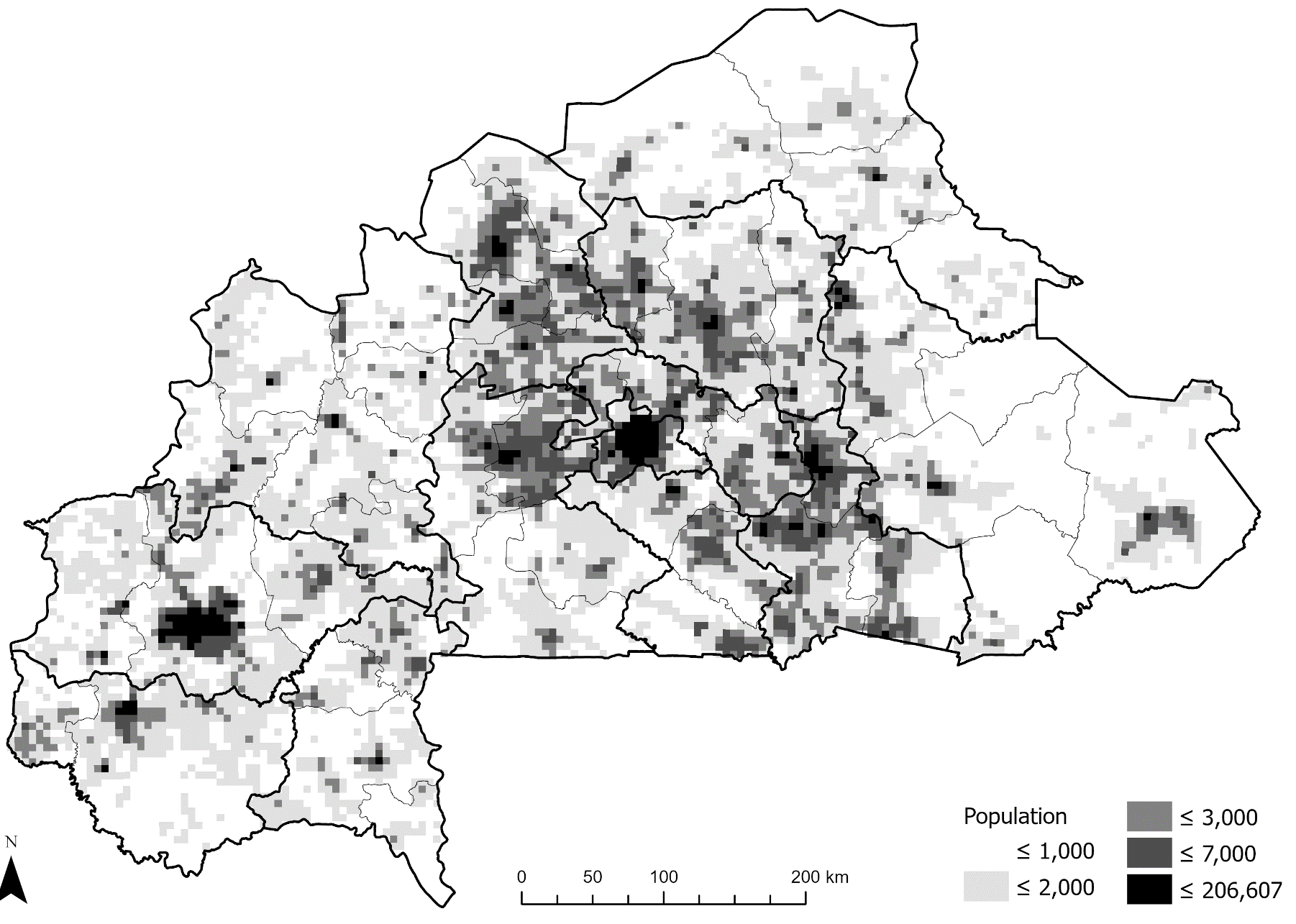


**References**

Marshall, J. M., Wu, S. L., Sanchez C., H. M., Kiware, S. S., Ndhlovu, M., Ouédraogo, A. L., … Ferguson, N. M. (2018). Mathematical models of human mobility of relevance to malaria transmission in Africa. *Scientific Reports*, *8*(1), 7713. doi: 10.1038/s41598-018-26023-1

World Bank Group. (2018). *Burkina Faso District Boundary*. ENERGYDATA.INFO. Retrieved from https://energydata.info/dataset/burkina-faso-administrative-boundaries-2017/resource/4215f2ee-ec6b-48b2-b725-97967e2e41f0

WorldPop. (2018). *Global High Resolution Population Denominators Project*. Retrieved from https://dx.doi.org/10.5258/SOTON/WP00645

Zupko, R. J., Nguyen, T. D., Somé, A. F., Tran, T. N.-A., Gerardin, J., Dudas, P., … Boni, M. F. (2022). Long-term effects of increased adoption of artemisinin combination therapies in Burkina Faso. *PLoS Glob. Public Health*, *2*(2), e0000111. doi: 10.1371/journal.pgph.0000111
